# Cyclodextrins Increase Triterpene Production in *Solanum lycopersicum* Cell Cultures by Activating Biosynthetic Genes

**DOI:** 10.3390/plants11202782

**Published:** 2022-10-20

**Authors:** Ana Belén Sabater-Jara, María Jesús Marín-Marín, Lorena Almagro, María Angeles Pedreño

**Affiliations:** Department of Plant Biology, Faculty of Biology, University of Murcia, Campus de Espinardo, E-30100 Murcia, Spain

**Keywords:** cyclodextrins, elicitation, gene expression, *Solanum lycopersicum* cell cultures, triterpenes

## Abstract

In this work, *Solanum lycopersicum* cv. Micro-Tom suspension-cultured cells were used to analyze the effect of different elicitors including β-cyclodextrins (CD), methyl jasmonate (MJ), β-glucan (Glu) and 3-hexenol (Hex) separately and the combined treatments of CD + MJ, CD + glu and CD + Hex on triterpene compound production after 24, 72 and 96 h. Moreover, we studied the changes induced by elicitors in the expression of key biosynthetic genes to elucidate the regulation of the triterpene biosynthetic pathway. The relative abundance of the triterpene compounds identified in the extracellular medium after elicitation (squalene, fucosterol, avenasterol, β-sitosterol, cycloartenol and taraxasterol) was determined by gas chromatography coupled to mass spectrometry, and the expression level of genes in treated-cells was analyzed by real-time quantitative polymerase chain reaction (qRT-PCR). Results showed that, in CD-treated cells (CD, CD + MJ, CD + Glu, CD + Hex), specialized metabolites were accumulated mainly in the extracellular medium after 72 h of elicitation. Moreover, qRT-PCR analysis revealed that the highest triterpene levels in CD-treated cells (CD, CD + MJ, CD + Glu, CD + Hex) were highly correlated with the expression of *cycloartenol synthase*, *3-hydroxy-3-methylglutaryl-CoA reductase* and *squalene epoxidase* genes at 24 h of treatment, whereas the expression of *sterol methyltransferase* was increased at 72 h. According to our findings, CD acts as a true elicitor of triterpene biosynthesis and can promote the release of bioactive compounds from the tomato cells into the extracellular medium. The results obtained provide new insights into the regulation of the triterpene metabolic pathway, which might be useful for implementing metabolic engineering techniques in tomato.

## 1. Introduction

*Solanum lycopersicum* (tomato) is a model plant used to study the development of its fruits and their production. Since the sequencing of its genome in 2012, *S. lycopersicum* has been widely used for genetic, genomic, developmental, and physiological studies, and new knowledge has been acquired through omics approaches [1,2,3]. Among the many existing tomato varieties, Micro-Tom (MT, a dwarf cultivar of tomato) is currently being used as a model cultivar because of its advantages over *Arabidopsis,* including its shorter life cycle and small size.

**Figure 1:**
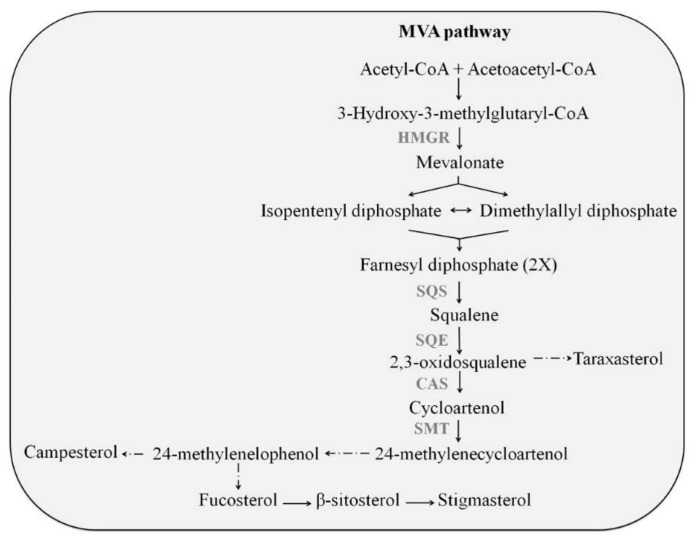
Simplified phytosterol biosynthetic pathway. HMGR: 3-hydroxy-3-methylglutaryl-CoA reductase; SQS: squalene synthase; SQE: squalene epoxidase; CAS: cycloartenol synthase; SMT: sterol methyltransferase.

Triterpenes have been extensively studied in the cuticle of tomato fruits, including compounds such as taraxasterol, lupeol, lanosterol, cycloartenol, cholesterol, stigmasterol and β-sitosterol [4,5,6,7,8]. Studies have also shown that different plant cell cultures exposed to elicitation can also produce phytosterols [9,10]. Phytosterols are part of the cell membranes in plants, and these compounds are involved in plant defense responses [11,12,13]. Triterpenes are isoprenoid compounds biosynthesized in the cytoplasm from farnesyl diphosphate (FPP) via the mevalonic acid (MVA) pathway [14]. Then, squalene synthase (SQS) can form squalene, the precursor of all sterols and other triterpenes in plants, from two FPP molecules [15]. The cyclization of squalene leads to the synthesis of 2,3-oxidosqualene in a reaction catalysed by squalene epoxidase (SQE) [16]. Depending on its spatial conformation, 2,3-oxidosqualene is converted to pentacyclic triterpenes (e.g., taraxasterol) for cyclization reactions [17] or to cycloartenol by cycloartenol synthase (CAS) [18] (Figure 1). Likewise, lanosterol synthase produces the cyclization product lanosterol in mammals, leading to cholesterol, and in fungi leading to ergosterol as end products [19].

Many of the triterpenic metabolites synthesized by *S. lycopersicum* are of particular interest because of their beneficial effects on human health. For example, they are reported to reduce stimulate blood circulation, maintain the lipid profile, remove toxins and decrease high blood pressure [20]. Thus, new environmentally friendly and sustainable methods have been developed for triterpene production, including elicited plant suspension-cultured cells (SCC) [21]. In our research group, we have obtained a system to produce these bioactive compounds based on the use of β-cyclodextrins (CD) in SCC [22]. CD can act as an elicitor, and it binds with apolar compounds, enhancing their release to the culture medium [23].

On the other hand, the elicitor which is most frequently used to enhance secondary metabolite production in plant in vitro cultures due to its effectivity is methyl jasmonate (MJ) (60% according to a literature search), followed by jasmonic acid and salicylic acid (approximately 10 and 15%, respectively) [24]. MJ is an essential oil derived from α-linolenic acid and was first isolated from *Jasminum grandiflorum* [25]. MJ regulates many physiological processes in plants, including development and growth as well as plant defense responses [26]. Instead, green leaf C6-volatiles ((Z)-3-hexenol (Hex), (Z)-3-hexenal and (Z)-3-hexenyl acetate) are mainly involved in allelopathic responses [27]. Furthermore, exposure of plant SCC to these volatile compounds was shown to induce the expression of genes related to defense responses and the production of phytoalexins [28]. Other used elicitors are β-glucans (Glu), oligosaccharides obtained from fungal cell walls [29]. Glu has been applied to trigger the biosynthesis of metabolites in soybean and rice cells [30,31] and the production of phytosterols and tocopherols in *Linum usitatissimum* SCC [23].

Considering these antecedents, the aim of the present work was to analyze triterpene biosynthesis in *S. lycopersicum* cv. Micro-Tom SCC after supplementing culture medium with the elicitors MJ, Glu and Hex, separately or in combination with CD. The capacity of these elicitors to regulate the biosynthesis and release of triterpene compounds in the culture medium was studied.

## 2. Results and Discussion

### 2.1. Effect of Elicitors on the Growth of MT Tomato SCC

**Figure 2:**
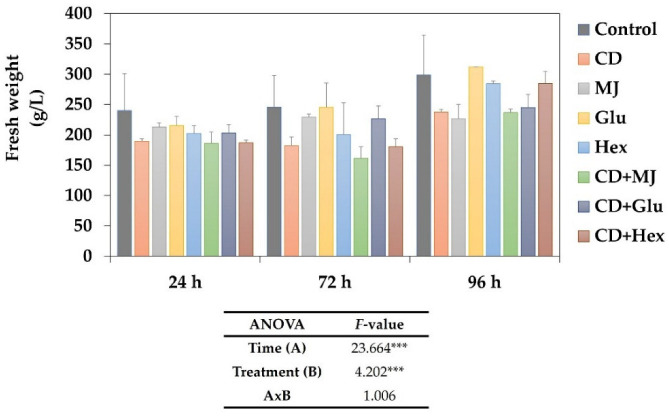
Effect of methyl-β-cyclodextrins (CD), alone or jointly with methyl jasmonate (MJ), glucan (Glu) or hexenol (Hex) on the cell growth of *S. lycopersicum* SCC expressed as g of fresh weight (FW) L^−1^ at 24, 72 and 96 h of treatment. F-values from two-way ANOVA significant at the 99.9% (***) level of probability.

In this study, the growth capacity of MT tomato SCC was evaluated for 96 h in SCC treated with 50 mM CD, 100 µM MJ, 1 mg L^−1^ Glu, and 40 µM Hex, alone or in combination (Figure 2). According to the ANOVA analysis, the growth of MT tomato SCC was significantly affected by the elicitor treatment and time of elicitation but not by the interaction between both factors. Regarding the elicitor treatments, the biomass accumulation was similar between the control and Glu, but when the culture medium was supplemented with CD, alone or in combination with MJ, a significant decrease in cell growth was detected. CD also had a slight negative impact on growth when applied with Glu or Hex. However, in all treatments, the maximum biomass accumulation was reached at 96 h of elicitation. Similarly, Vidal-Limón et al. [28] found reduced biomass in *Taxus media* SCC in the presence of CD + coronatine; this effect was even more pronounced when the *T. media* cells were elicited with Hex + CD + coronatine. In contrast, Almagro et al. [23] described no negative effect on cell growth of *L. usitatissimum* SCC elicited with Glu or Hex or in the combined treatments with CD. On the other hand, several studies have reported that MJ causes a modification of secondary metabolism and arrests the cell cycle in SCCs of some plant species [32,33]. The reduction in cell growth associated with elicitation arises from competition between the defense- and growth-related metabolism [34,35]. In fact, to survive under stress conditions, plants must control cell growth patterns, regulate cellular redox homeostasis, maintain cellular metabolic functions, and activate secondary metabolism [36].

### 2.2. Effect of Elicitors on Total Triterpene Content in MT Tomato SCC

**Figure 3:**
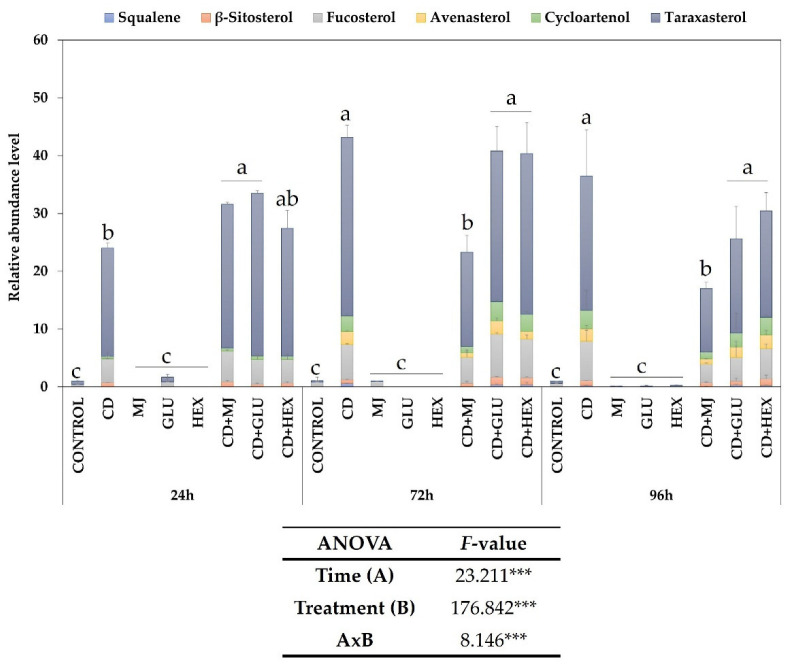
Relative abundance level of the triterpenoid compounds identified in the extracellular medium of *Solanum lycopersicum* SCC treated with methyl-β-cyclodextrins (CD), alone or jointly with methyl jasmonate (MJ), glucan (Glu) or hexenol (Hex) at 24, 72 and 96 h of treatment. The relative abundance levels have been presented as the fold increase relative to the control. Bars with different letters show significant differences (*p* < 0.05) between the treatments used within each time according to Tukey’s test (*p* ≤ 0.05). *F*-values from two-way ANOVA significant at the 99.9% (***) level of probability.

It is well-known that elicitors can induce the production of primary and secondary metabolites in some plant SCC [23]. As well as acting as a true elicitor, CD form inclusion complexes with apolar metabolites, which favors their release into the extracellular medium [37]. The application of CD at a concentration of 50 mM has been described as optimal for both actions [22]. Here, the concentrations of MJ, Glu and Hex were selected based on our previous data [9,10,23]. Hence, we analyzed the effect of 50 mM CD, applied alone or jointly with 1 mg L^−1^ Glu, 100 µM MJ, and 40 µM Hex, on the production of triterpenes in MT tomato SCC (Figure 3). For this purpose, the extracellular medium was sampled periodically at 24, 72 and 96 h after elicitation. As shown by the ANOVA analysis, both the treatment type and duration, as well as the interaction of these two factors, significantly influenced triterpene production (Figure 3). Thus, a significant enhancement in total triterpenes was observed after MT tomato SCC were treated with CD, with the maximum extracellular levels being reached at 72 h of elicitation with 50 mM CD, alone or jointly with Glu, Hex or MJ (Figure 3). Of the six triterpenes identified by GC-MS in the extracellular medium, the most abundant were taraxasterol, which represented more than 60% of the triterpenes identified, followed by fucosterol (around 17%), and to a lesser extent, squalene, β-sitosterol, avenasterol and cycloartenol were also found (Figure 3). The intracellular levels of total triterpenes were very low in all treatments, as in the control cells (data not shown).

According to these results, CD increased the levels of triterpenes in the extracellular medium due to their structural properties since they can trap triterpenes in their hydrophobic central cavity, forming an inclusion complex [38]. By isolating the metabolites, CD can protect them from degradation and enhance their release into the culture system [36]. Previously, it was reported that elicitation with CD improved triterpene production in SCC of tomato, carrot, and flax, respectively [10,23,39]. On the other hand, Sharma and Zafar [40] showed that treating *Taraxacum officinale* Weber cultures with 25 mM CD or 200 µM MJ increased the production of taraxasterol, as in our experiments. The enhanced extracellular triterpene accumulation demonstrated in the present study indicates that CD-elicited MT tomato SCC constitutes a highly promising biotechnological production system for bioactive compounds of pharmacological interest.

### 2.3. Effect of Elicitors on the Expression of Triterpene Biosynthetic Genes

**Figure 4:**
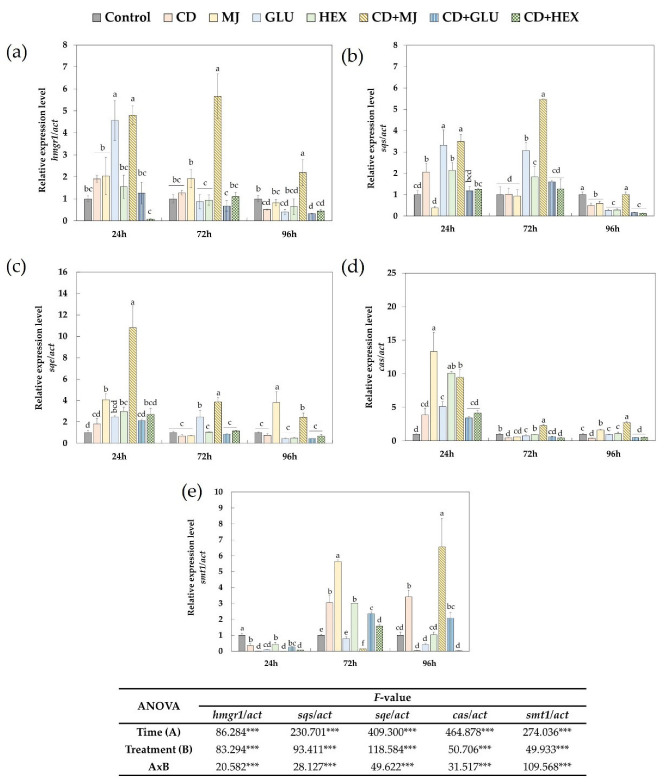
Relative expression level of *hmgr1* (**a**), *sqs* (**b**), *sqe* (**c**), *cas* (**d**) *and smt1* (**e**) genes in *S. lycopersicum* SCC elicited with methyl-β-cyclodextrin (CD), alone or jointly with methyl jasmonate (MJ), glucan (Glu) or hexenol (Hex) at 24, 72 and 96 h of treatment. The control with reference value = 1 was used to normalize the relative expression levels of each gene. Levels of transcripts were calculated using the actin gene as internal control. Bars with different letters show significant differences (*p* < 0.05) between the treatments used within each time according to Tukey’s test. *F*-values from two-way ANOVA significant at the 99.9% (***), evel of probability.

Having established that the elicitation of MT tomato SCC with CD, individually or jointly with MJ, Glu or Hex, triggers a significant accumulation of triterpenes (mainly fucosterol and taraxasterol) in the culture medium (Figure 3), we analysed the expression of key biosynthesis genes by qRT-PCR to identify how the presence of elicitors affected to the triterpene biosynthesis (Figure 4). The genes studied were: *hmgr1* (encoding 3-hydroxy-3-methylglutaryl coenzyme A reductase 1 (HMGR1), Figure 4a), *sqs* (squalene synthase (SQS), Figure 4b), *sqe* (squalene epoxidase, Figure 4c), *cas* (cycloartenol synthase, Figure 4d), and *smt1* (S-adenosyl-2-methionine:(24 (28) C-methyltransferase 1 (SMT), Figure 4e). According to the ANOVA analysis, expression levels of all target genes were significantly influenced by the duration and type of treatment, as well as by the interaction of both factors. However, all elicitors tested triggered different gene expression patterns throughout the experiments (from 24 to 96 h).

The enzyme HMGR1 catalyses the biosynthesis of MVA produced by HMG-coenzyme A (Figure 1). This reaction represents a limiting step in the triterpene biosynthetic pathway [41] and is sensitive to multiple stimuli, both external (light, wounds, etc.) and internal (e.g., phytohormones). As shown in Figure 4, the maximum transcript levels of *hmgr1* were detected at 24 h of incubation with Glu (Figure 4a), when they were about 5-fold higher than in control cells. Furthermore, it should be noted that only the combined treatment of CD + MJ induced a significant up-regulation of *hmgr1* until 72 h when the expression was 4.4- and 2.9-fold higher than in cells incubated with CD or MJ individually (Figure 4c), and never, the combined treatment of Glu or Hex with CD, increased the expression levels of *hmgr1* above the control cells.

The relative expression levels of *sqs* encoding the sterol and triterpene pathway-specific SQS (Figure 1) in MT tomato SCC treated with CD, MJ, Glu or Hex, alone or in combination, at 24, 72 and 96 h of elicitation are depicted in Figure 4b. Elicitation with Glu significantly increased *sqs* transcript accumulation at 24 h (3-fold with respect to control cells), which subsequently decreased until 96 h. Hex also increased the expression of *sqs* at 24 h but to a lesser extent than Glu. Similar to *hmgr1*, the *sqs* gene was significantly upregulated by the combination of CD + MJ, its expression being around 5.5-fold higher compared to the control treatment at 72 h of incubation.

Expression of the *sqe* gene was also studied (Figure 4c). Its maximum expression was found at 24 h of treatment with CD + MJ (10.8-, 6- and 2.6-fold higher than in control, CD- and MJ-treated cells, respectively). The transcript levels of *sqe* were slightly increased by treatments with CD, CD + Glu or CD + Hex at 24 h, otherwise remaining quite constant during the study period.

The expression of the *cas* gene, the enzyme responsible for generating cycloartenol (Figure 4d), increased significantly in all treatments at 24 h of elicitation. The highest increases were detected after elicitation with MJ, individually or in combination with CD (CD + MJ) and Hex (13-, 9.5- and 10-fold higher, respectively, than the control). As mentioned, eukaryotic triterpene biosynthesis begins with 2,3-oxidosqualene, which leads to the formation of pentacyclic triterpenes such as taraxasterol or generates cycloartenol, the precursor of all the known phytosterols. As the relative abundance of taraxasterol was double that of total phytosterols (cycloartenol + β-sitosterol + fucosterol), these results suggest that CD, individually or with MJ, Hex or Glu, activates the expression of the *cas* gene and could induce the putative genes involved in taraxasterol biosynthesis.

The last gene analysed from the phytosterol biosynthetic pathway was *smt1* (Figure 4e), which encodes SMT, the enzyme that methylates carbon 24 of cycloartenol to obtain 24-methylcycloartenol (Figure 1). The expression levels of *smt1* (Figure 4e) were always lower in all treated cells compared to the control at 24 h of elicitation. In contrast, the maximum transcript levels were observed in cell cultures incubated with MJ alone (at 72 h) or with CD (at 96 h), being on average 5.6- and 6.5-fold higher than in the control, respectively. CD and Hex alone also increased the expression of *smt1* at 72 h of treatment, whereas Glu had no impact. The upregulation of *smt1* in MT tomato SCC treated with CD, alone or with MJ, Glu or Hex, at 72 h of elicitation correlated well with the increment in the relative abundance of fucosterol, β-sitosterol, avenasterol and cycloartenol in the extracellular medium at 96 h of treatment (Figure 3).

Therefore, the enhanced extracellular accumulation of triterpenes observed in MT tomato SCC elicited with CD, individually or jointly with MJ, Glu or Hex, correlated well with the higher expression levels of *hmgr1*, *sqe*, *cas* and *smt1* at any of the times analysed. The results indicate that CD can act as an elicitor, promoting both the biosynthesis of triterpenes as well as their excretion from the cells, which favours their accumulation in the extracellular medium. Miras-Moreno et al. [42] also observed an enhancement in the expression of the *cas* but not the *sqs* gene in CD-treated *Daucus carota* SCC. Similarly, in *D. carota* SCC, elicitation with CD increased the extracellular accumulation of phytosterols [39].

## 3. Materials and Methods

### 3.1. Plant Materials

Friable calli from *Solanum lycopersicum* L. cv. Micro-Tom (MT tomato) were obtained from fruits of tomato in vitro plants, and they were maintained at 25 °C under a 16 h light/8 h dark photoperiod with a photon flux density of 85 µmol m^−2^ s^−1^ at 25 ºC. These calli were subcultured every 21 days on a solid Murashige and Skoog [43] basal medium supplemented with Morel vitamins [44], 0.8 mg L^−1^ naphthalenacetic acid, 0.1 mg L^−1^ kinetin, 0.25 g L^−1^ casein hydrolysate, and 30 g L^−1^ sucrose at pH 5.8. SCC were established by transferring 20 g of friable callus into 250-mL Erlenmeyer flasks containing 100 mL of the same medium without agar, and they were maintained in the same conditions of tª and photoperiod as described above in an orbital shaker at 115 rpm. These MT tomato SCC were subcultured every 15 days by diluting the cells with an equal volume of culture medium.

### 3.2. Elicitation of MT Tomato SCC

Elicitation treatments were applied to 15-day-old MT tomato SCC. The treatments were 50 mM β-CD (Wacker Chemie, Madrid, Spain), 100 µM MJ (Duchefa Biochemie, Haarlem, The Netherlands, 40 μM *cis*-3-hexen-1-ol (Hex; Sigma-Aldrich, Madrid, Spain), 1 mg L^−1^ Glu (Sigma-Aldrich, Madrid, Spain) and the combined treatments of CD plus MJ (CD + MJ), Glu (CD + Glu) and Hex (CD + Hex). Briefly, 4 g of fresh weight (FW) of MT tomato cells from SCC were grown in 20 mL sterile fresh medium containing CD alone or with MJ, Hex or Glu, the quantities being based on previous experience [5,6,13,14]. After 24, 72 and 96 h of incubation, the culture medium was separated from the cells by gentle vacuum filtration. Triterpene metabolites were extracted from the culture medium, the extracts were analysed by GC-MS as described by Miras-Moreno et al. [45], and the cells were used to extract RNA to analyse gene expression. 

### 3.3. Extraction and Identification of Triterpene Compounds

Triterpene metabolites were extracted for aqueous two-phase partition with ethyl acetate (1:1, *v*/*v*). The organic phase was collected and evaporated at 40 °C in vacuum; the dry extract was dissolved in 1 mL methanol for the chromatographic analysis. Identification of triterpene compounds was based on mass spectra obtained by a gas chromatograph Agilent Technologies 6890 Network GS System equipped with a mass selective detector in a capillary column 30 m × 0.25 mm (Agilent 19,091 S-433HP-5MS) (GC-MS). A range of temperature from 60 to 310 °C was programmed as the GC oven temperature for the analysis of triterpenes. Helium was used as carrier gas at 0.1 mL min^−1^. An ionization energy of 70 eV was used, and the mass range was recorded from *m*/*z* 50–800. The injection volume was 1.0 μL. The results were analysed using Chemstation software. The identification of the triterpene compounds in the extracellular medium was carried out by comparing the experimental mass spectra and retention time with those obtained from commercial external standards and those that appear in the NBS 75 K library. The results were expressed as the sum of the relative abundance of each identified compound. All experiments were performed in triplicate.

### 3.4. Quantitative Real-Time RT-PCR (qRT-PCR)

The total RNA was extracted from 500 mg cells using a Trizol reagent (Invitrogen, Madrid, Spain) following the manufacturer’s protocol. The purification included a DNase treatment using the RNase free DNase Set (Qiagen, Hilden, Germany). The yield and purity of the RNA were determined using a NanoDrop ND-2000 spectrophotometer (Thermo Fisher Scientific, Waltham, MA, USA). Starting from 1 µg of the purified RNA, the cDNA was synthetized using the RevertAid First Strand cDNA Synthesis Kit (Thermo Fischer Scientific, Waltham, MA, USA) according to the manufacturer’s protocol. The cDNA samples obtained were kept at −80 °C until used. qRT-PCR was performed in an Applied Biosystems QuantStudio 7500 Real-Time PCR system (Thermo Fisher Scientific, Waltham, MA, USA) in 10 μL, using the 2X Power SYBR Green PCR Master Mix (Applied Biosystems, Carlsbad, CA, USA). Here, we used 5 μL SYBR Green reaction mix, 51 nM forward and reverse gene-specific primers and 33 ng of cDNA. Gene-specific primers were designed with OligoAnalyzer 3.1 software (Table 1). The reaction conditions and the efficiency of each primer pair were determined by performing a calibration curve with serial dilutions from a pool of all cDNA samples, as described by Qiagen (Hilden, Germany). For each gene, expression values were normalized to actin (*act*) as the reference gene. The relative expression values of the *hmgr1, sqs, sqe, cas* and *smt1* genes were analyzed after 24, 72 and 96 h of elicitation. All experiments were performed in triplicate.

### 3.5. Statistical Analysis

Data were expressed as the mean ± standard deviation (SD) of three independent replicates. For comparing the treatment means, an analysis of variance (ANOVA) was tested by Tukey’s honestly significant difference (HSD) test using the Statistical Package for the Social Sciences software version 22 (SPSS Inc., Chicago, IL, USA), and statistically significant differences were considered at *p* < 0.05.

## 4. Conclusions

The work reported here involved an in-depth study of triterpene biosynthesis, especially taraxasterol and phytosterols in MT tomato SCC. Elicitation with CD, alone or jointly with MJ, Glu, or Hex, after 96 h of treatment increased the extracellular accumulation of triterpenes, especially of the phytosterol, fucosterol, and the pentacyclic triterpene taraxasterol. Moreover, the comparative analysis of the metabolomic and transcriptomic profile in elicited MT tomato SCC showed that the expression of key genes of triterpene biosynthesis correlated well with the extracellular levels of fucosterol and taraxasterol. However, although the maximum transcript levels were reached at the beginning of the elicitation (24 h), the highest accumulation of triterpenoids occurred at 96 h of treatment. Therefore, in this plant system, CD acts not only as a sequester of highly apolar metabolites in the culture medium but also as a true elicitor, as it induced the upregulation of genes related to plant defense. This work shows that elicitors could be used to modulate bioactive compound levels in food plants like tomatoes.

## Figures and Tables

**Table 1:** List of specific primers used for qRT-PCR.

Gene Abbreviation	Accession Number	Primer Pair (5′-Forward-3′/5′-Reverse-3′)	Size (pb)
***hmgr1***	NM_001309881.1	CTTCCACTCCCATTGTACCTAAC/GATCTTCTCACGCCACCTTAC	93
***sqs***	NM_001247787.2	ACCCACCGATGTTAAAGTACC/CTGGTCCATGAGAACCTTGT	108
***sqe***	XM_026029244.1	GCACATGCTCCTCTTACAGTAG/GAGGGAACATCAACCTTAGGG	86
***cas***	NM_001246855.2	CGCTTTGTTGGTCCTATCACT/GAGGGTGTGGGTAGTAAAGG	133
***smt1***	XM_004229602.3	AAGTTCTCTCTGCTGTTGACAAA/ACTCTCCCCATCCGTATTCATAGAAG	144
***act***	FJ532351.1	TCAGGCTGTGCTTTCCTTGT/CGACCAGCAAGATCCAAACG	141

## Data Availability

The data is contained within the article.
